# The Effects of Tai Chi on Type 2 Diabetes Mellitus: A Meta-Analysis

**DOI:** 10.1155/2018/7350567

**Published:** 2018-07-05

**Authors:** Mengyao Chao, Chunyan Wang, Xiaosheng Dong, Meng Ding

**Affiliations:** College of Physical Education, Shandong Normal University, 88 Wenhuadong Street, Jinan 250014, China

## Abstract

**Objective:**

To investigate the effects of Tai chi in type 2 diabetes mellitus (type-2 DM) patients using systematic review and meta-analysis.

**Methods:**

Seven electronic resource databases were searched, and randomized controlled trials on the role of Tai chi in type-2 DM patients were retrieved. The meta-analysis was performed with RevMan 5.3, and research quality evaluation was conducted with the modified Jadad scale.

**Results:**

Fourteen studies, with 798 individuals related to the intervention of Tai chi on diabetes, were included. The results showed that, compared with nonexercise, Tai chi had the effect of lowering fasting blood glucose [MD = −1.39, 95% CI (−1.95, −0.84), *P* < 0.0001] and the subgroup effect size decreased with the increase of total exercise amount, there is no significant difference between Tai chi and other aerobic exercises [MD = −0.50, 95% CI (−1.02, 0.02), *P* = 0.06]; compared with nonexercise, Tai chi could reduce HbA1c [MD = −0.21, 95% CI (−0.61, 0.19), *P* = 0.31], and the group effect size decreased with the increase of total exercise amount. The reducing HbA1c effect of Tai chi was better than that of other aerobic exercises, but the difference was at the margin of statistical significance [MD = −0.19, 95% CI (−0.37, 0.00), *P* = 0.05]; compared with nonexercise, Tai chi had the effect of reducing 2 h postprandial blood glucose [MD = −2.07, 95% CI (−2.89, −1.26), *P* = 0.0002], there is no significant difference between Tai chi and other aerobic exercises in reducing 2 h postprandial blood glucose [MD = −0.44, 95% CI (−1.42, 0.54), *P* = 0.38].

**Conclusion:**

Tai chi can effectively affect the management of blood glucose and HbA1c in type-2 DM patients. Long-term adherence to Tai chi has a better role in reducing blood glucose and HbA1c levels in type 2 DM patients.

## 1. Introduction

Diabetes mellitus (DM) is a chronic disease characterized by chronic hyperglycemia, which triggers the changes in lipids and proteins. There are 425 million people with diabetes in the world. There will be 629 million people with diabetes in the world in 2045 [1]. Diabetes has become the third major noncontagious disease threatening human health in modern countries, only second to cardiovascular disease and cancer [2].

According to the latest statistics, there were about 110 million adults suffering from diabetes in China, and it had rapidly become one of the countries with the medium prevalence (3%–10%) from the previous one with low prevalence (<3%) in less than 20 years [3, 4]. At present, the total cost of medical treatment due to diabetes and its complications in China was 20.86 billion yuan per year, accounting for 4.38% of the total national medical expenses, resulting in tremendous personal, family, and socioeconomic burdens [5, 6].

It is believed that the improvement of lifestyles can effectively control and prevent the occurrence of type-2 DM and its complications. The American Diabetes Association (ADA) pointed out that exercise therapy was an important part of improving lifestyle: effective exercise could prevent and control type-2 DM, playing a positive role in lowering blood glucose [7]. For example, moderate-intensity aerobic exercise and appropriate resistance exercise had an improving effect on blood glucose and blood lipid indicators of type-2 DM. Aerobic exercise could ameliorate the levels of visceral fat area and HbA1c in middle-aged and elderly obese patients with type-2 DM and improve the lipid metabolism, body composition, and cardiopulmonary function at the same time; accordingly, the blood glucose level was effectively controlled and the incidence of type-2 DM was reduced. In summary, exercise therapy has become an important part of the first level intervention of diabetes, which contributes to the management of type-2 DM patients [8].

The pathogenesis of type-2 DM is closely related to individual's physical and mental state. The traditional exercise, Tai chi, is a combination of physical activity and respiration, which combines limb movement and mind, making people enjoying physical and mental pleasure while exercising [9]. Therefore, Tai chi would play a more effective role in maintaining the body's neuroendocrine balance and controlling blood glucose. Although some researchers have done some works on the effects of Tai chi in type-2 DM patients using systematic evaluation and meta-analysis [10, 11], there are some inadequacies, including limited number of subjects and not typical enough detection indicators. In addition, as a form of physical and mental integration, whether Tai chi have more advantages beyond other aerobic exercises in the management of type-2 DM still lacks relevant merged research evidence. Accordingly, it is necessary to further confirm the effects of Tai chi on type-2 DM by including more high-quality studies. Besides, although some studies have explored the dose-response relationship between exercise and the incidence of type-2 DM using meta-analysis [12, 13], whether exercise, including Tai chi, had a dose-response relationship with blood glucose control was rarely studied. Therefore, this study also intends to preliminarily investigate the dose-response relationship between the exercise amount of Tai chi and blood glucose lowering in type-2 DM patients using meta-analysis.

## 2. Materials and Methods

### 2.1. Data Sources and Search Strategies

Electronic resource databases were searched, including PubMed, The Cochrane Library, EM base, SCI, CNKI, Wanfang Data, and VIP, and randomized controlled trials on the intervention effect of Tai chi on diabetes were retrieved. The time was all set as from the beginning to June 2016. Meanwhile, the references of the included papers were also reviewed to avoid omission. The search terms were divided into two parts: target (diabetes) search and intervention measure (Tai chi) search, and adjusted according to each database. All the searches used a combination of thematic and free search; all search strategies were determined after multiple presearch. The search strategy was mainly based on advanced search terms, including Tai chi, Taiji, Taijiquan, diabetes, type-2 diabetes mellitus, and DM.

### 2.2. Inclusion and Exclusion Criteria

Inclusion criteria included randomized controlled trials, published in Chinese and English language, with no restrictions on age or gender of type-2 DM; experimental group that takes Tai chi as the major intervention in the exercise of type-2 DM; the control group that does not take any exercise or other aerobic exercises (aerobic exercise except traditional sports including Tai chi and Qigong: jogging, walking, etc.) or antiresistance exercises; and patients without serious DM-related complications.

Exclusion criteria included experimental groups that used voluntary grouping principles and other nonrandomized controlled experiments; studies that did not include indicators to be discussed in this study; experimental groups that used other main exercises excluding Tai chi; and patient with DM-related complications.

### 2.3. Trial Inclusion and Data Extraction

During the process, two researchers (M. C. and X. D.) independently completed the trial inclusion and data extraction, and in case of disagreements, the third researcher (M. D.) would determine whether to include and extract. Data extraction information included information of the first author, publication time, published journals, title of trials, intervention measures of the experimental group and the control group, intervention time, subject number of the experimental group and the control group, whether outcome indicator was complete, whether there were subjects lost to follow-up, and basic information of patients (age, gender, sex ratio, and whether there were other complications). Diabetes indicator data included fasting blood glucose (FBG), HbA1c, and 2 h postprandial blood glucose (2hPBG); FBG was the first indicator of this study.

### 2.4. Trial Quality Assessments

During the data extraction process, the quality of the included studies was evaluated using modified Jadad quality scoring scale [14]. Modified Jadad quality scoring scale included the generation of random sequences, distribution methods, randomized concealment, whether blind method was adopted, and so on. There were corresponding criteria and scores for each item, 1–3 points for low-quality study and 4–7 points for high-quality study. Two raters performed the quality assessment independently (M. C. and C.W.). Disagreements were resolved by seeking the opinion of the third rater (M. D.)

### 2.5. Statistical Analysis

Meta-analysis was performed with Review Manager 5.3 available from Cochrane. The heterogeneity test *χ*^2^ of the results was included. When there was statistical homogeneity between studies, it was shown in *P* > 0.1 and *I*^2^ < 50% and a fixed-effect model was used; the heterogeneity was shown in *P* < 0.1 and *I*^2^ > 50%, and heterogeneity source analysis was performed, then subgroup analysis was conducted based on the heterogeneity. If there was no heterogeneity between subgroups, a fixed-effect model was used, while if there was heterogeneity between subgroups, the meta-analysis was performed with a random-effect model; if the heterogeneity within the group was too large, descriptive analysis was carried out.

To reveal whether there was a dose-response relationship between Tai chi with blood glucose control and HbA1c lowering in type-2 DM patients, Tai chi and nonexercise in the included studies were compared based on total exercise amount, and stratified subgroup analysis was performed in this study. Since most of the selected studies did not report Tai chi exercise intensity, the exercise intensity of all selected studies was treated as the same by default; thus, the total exercise amount could be expressed by total exercise time. According to total exercise amount, the included studies were divided into 4 subgroups, namely, small exercise group (with exercise amount less than 1999 minutes), medium exercise group (with exercise amount of 2000–3999 minutes), large exercise group (with exercise amount of 4000–5999 minutes), and super large exercise group (with exercise amount above 6000 minutes). The total exercise amount was calculated as total exercise amount (minutes) = Tai chi exercise time (minutes) per day ^∗^ Tai chi exercise days per week ^∗^ total of Tai chi exercise weeks (calculated as 4 weeks per month).

## 3. Results

### 3.1. Trial Search

**Figure 1:**
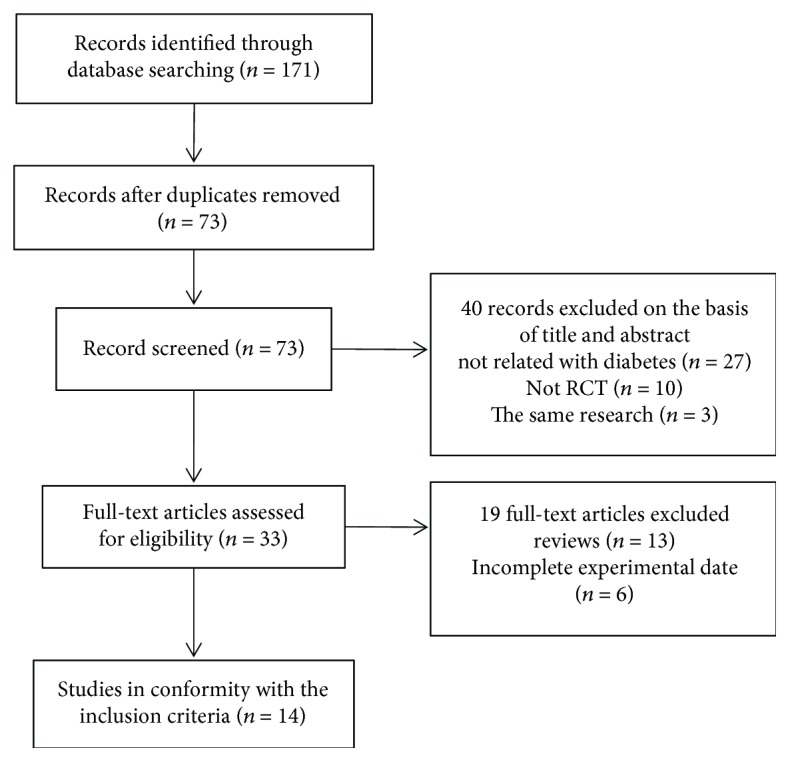
Flowchart of trial selection process.

Databases, including PubMed, The Cochrane Library, EMbase, SCI, CNKI, Wanfang Data, and VIP, were searched, and according to the criteria of exclusion and inclusion, randomized controlled trials on the intervention of Tai chi on type-2 DM were retrieved. A total of 171 potentially relevant articles were identified by the database searches. After excluding duplicates, 73 records remained. Of these, 40 articles were removed through reading titles and abstracts and 19 were excluded after reviewing the full text against the eligibility criteria. Finally, a total of 14 trials [15–27], including 5 English ones and 9 Chinese ones, were included. One trial titled “Health Qigong Tai Ji Quan Adjuvant Therapy in Patients with Type 2 Diabetes Clinical Observational Studies” is an unpublished literature, which was retrieved from the Doctoral and Master Dissertation Library of CNKI. The study selection process is shown in Figure 1.

### 3.2. Characteristics of Included Studies and Quality Assessments

Fourteen studies, with 798 individuals related to the intervention of Tai chi on diabetes, were included. The average age ranged 48.0–64.0. All patients in intervention groups received Tai chi intervention while maintaining routine medication. Seven articles have adopted the 24-style (short form) Tai chi [17, 18, 22, 23, 25, 27, 28], 1 article adopted the 24-form Yang TC style [19], 1 article adopted Yang and Sun 20-form TC style [20], 1 article adopted the Lin Housheng's style [24], 1 article adopted the DaYuan's TC style [15], 1 article adopted the Yang TC style [21], and 2 articles did not report the type of Tai chi [16, 26]. The frequencies of Tai chi exercise in the included articles were from 3 to 7 times per week, and exercise durations lasted from 15 to 60 minutes across the different articles. The control groups were divided into nonexercise groups and other aerobic exercise groups. The exercise forms of other aerobic exercise groups include social dance, Qigong, and general aerobic exercise (see Table 1 for details). In the case of modified Jadad scoring, there were 6 studies with 2 points, 5 studies with 3 points, 2 studies with 5 points, and 1 study with 6 points. There were 11 low-quality trials, with an average of 2.4 points, and 3 high-quality trials, with an average of 5.3 points (see Table 2 for details).

### 3.3. Outcomes

#### 3.3.1. Fasting Blood Glucose

**Figure 2:**
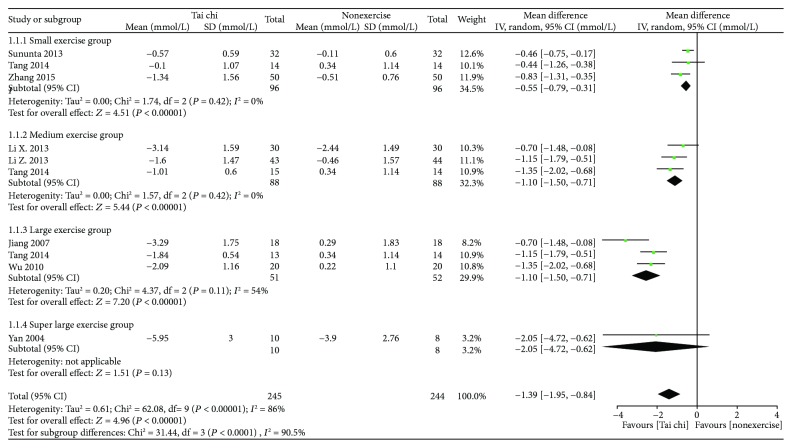
The meta-analysis for comparing FBG between Tai chi and the nonexercise. CI: confidence interval; SD: standard deviation.

*(1) Tai Chi versus Nonexercise*. Initially, the data of 11 related studies (*n* = 529) [15, 17, 23–27] was pooled, but the data heterogeneity was quite high. After subgroup analysis according to total exercise amount, it was found that the heterogeneity of 4 studies in a large exercise group was as high as 94%. Through sensitivity analysis of the data, it was speculated that the data of Yu 2004 was the main source of heterogeneity in this subgroup. Accordingly, the data of this study was excluded, and the data of the other 3 studies was pooled. Finally, the data of 10 studies (*n* = 489) was pooled, and the results showed that, compared with nonexercise, Tai chi had an effect of lowering FBG and that the difference was statistically significant [MD = −1.39, 95% CI (−1.95, −0.84), *P* < 0.0001]. Within 6000-minute exercise amount, the subgroup pooled effect size decreased with the increase of total exercise amount, and only one study was included above 6000 minutes, its effect size was close to the group of 4000–5999 minutes and the difference was not statistically significant (see Figure 2).

**Figure 3:**
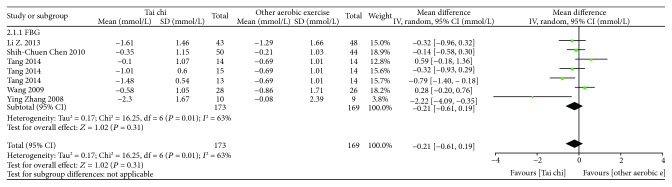
The meta-analysis for comparing FBG between Tai chi and other aerobic exercises. CI: confidence interval; SD: standard deviation.

*(2) Tai Chi versus Other Aerobic Exercises*. As shown in Figure 3, 7 related studies (*n* = 342) [18, 21, 22, 25] allowed for data pooling. After data pooling using a random-effect model, the results suggested that, compared with other aerobic exercises, Tai chi did not show a better role in lowering FBG, and the difference was not statistically significant [MD = −0.21, 95% CI (−0.61, 0.19), *P* = 0.31].

#### 3.3.2. HbA1c

**Figure 4:**
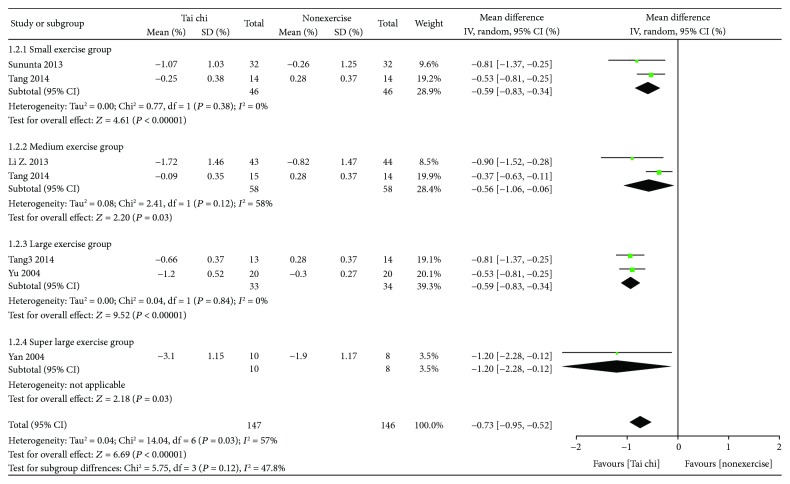
The meta-analysis for comparing HbA1c between Tai chi and the nonexercise. CI: confidence interval; SD: standard deviation.

*(1) Tai Chi versus Nonexercise*. Initially, the data of 9 studies (*n* = 356) [15, 16, 24, 25] was pooled, but the data heterogeneity was quite high. After subgroup analysis according to total exercise amount, it was found that the heterogeneity was mainly from the large exercise group. After sensitivity analysis, it was speculated that the data of Jiang 2007 and Wu 2010 was the main source of heterogeneity. Accordingly, the data of these two studies was excluded, and data pooling was reperformed. Finally, data pooling was carried out for 7 studies (*n* = 293), and the results showed that HbA1c in the Tai chi group was lower than that in the control group, with statistically significant difference [MD = −0.73, 95% CI (−0.95, −0.52), *P* = 0.03]. The grouping effect size of HbA1c decreased with the increase of total exercise amount (see Figure 4).

**Figure 5:**
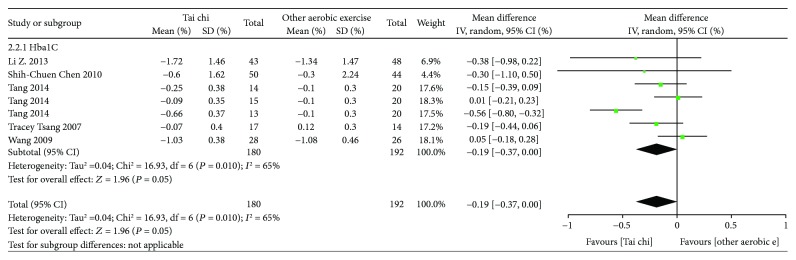
The meta-analysis for comparing HbA1c between Tai chi and other aerobic exercises. CI: confidence interval; SD: standard deviation.

*(2) Tai Chi versus Other Aerobic Exercises*. As shown in Figure 5, 7 related studies (*n* = 372) [19, 21, 22, 25] allowed for data pooling. After data pooling using a random-effect model, the results suggested that, compared with other aerobic exercises, Tai chi had a better effect of reducing HbA1c and that the difference was statistically significant [MD = −0.19, 95% CI (−0.37, 0.00), *P* = 0.05]. However, the statistical significance was at the margin.

#### 3.3.3. Two-Hour Postprandial Blood Glucose

**Figure 6:**
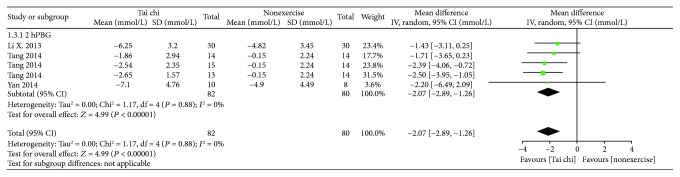
The meta-analysis for comparing 2hPBG between Tai chi and the nonexercise. CI: confidence interval; SD: standard deviation.

*(1) Tai Chi versus Nonexercise*. As shown in Figure 6, 5 related studies (*n* = 162) [15, 26] allowed for data pooling. After data pooling using a random-effect model, the results suggested that the 2hPBG in the Tai chi group was lower than that in the nonexercise group and that the difference was statistically significant [MD = −2.07, 95% CI (−2.89, −1.26), *P* = 0.0002].

**Figure 7:**
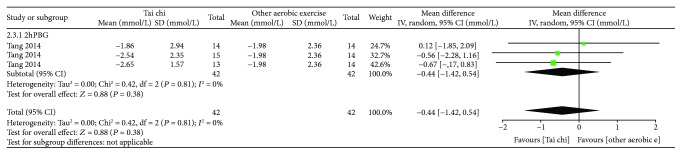
The meta-analysis for comparing 2hPBG between Tai chi and other aerobic exercises. CI: confidence interval; SD: standard deviation.

*(2) Tai Chi versus Other Aerobic Exercises*. As shown in Figure 7, for 3 related studies (*n* = 84), after data pooling using a random-effect model, the results showed that there was no statistically significant difference in 2hPBG between the Tai chi group and the other aerobic exercise group [MD = −0.44, 95% CI (−1.42, 0.54), *P* = 0.38].

### 3.4. Publication Bias Evaluation

#### 3.4.1. Publication Bias Evaluation between Tai Chi and Nonexercise

In this study, funnel plot analysis was performed on the indicator, FBG, in the Tai chi group and the nonexercise group. A total of 11 studies, with 529 subjects, were included. The results showed that the distribution of included studies on both sides of the funnel plot was asymmetric, indicating that there might be potential publication bias in the comparison of Tai chi and nonexercise.

#### 3.4.2. Publication Bias Evaluation between Tai Chi and Other Aerobic Exercises

Funnel plot analysis was performed on the indicator, FBG, in the Tai chi group and the other aerobic exercise group. A total of 7 studies, with 342 subjects, were included. The results showed that the distribution of included studies on both sides of the funnel plot was asymmetric, indicating that there might be potential publication bias in the comparison of Tai chi and other aerobic exercises.

## 4. Discussion

Tai chi is one of the unique traditional exercises in China. In the practice of Tai chi, it is required to be concentrated, move slowly, and be tranquil in movement. The intensity is not so high, and adequate physical and mental relaxation can be achieved. Thus, Tai chi is fit for older people with weaker constitution and chronic diseases, which is a moderate general movement. Theoretically, Tai chi should be a suitable exercise for type-2 DM patients. However, there were contradictions in published systematic review and meta-analysis. Zhang Yeting and others included 4 studies and systematically reviewed the effect of Tai chi in type-2 DM patients before and after exercise; the results showed that there was a significant decrease of blood glucose level in type-2 DM patients after Tai chi exercise, indicating the effect of Tai chi on diabetes, while two meta-analysis researches published in 2013 and 2015 [10, 11] considered that there was still no sufficient evidence to confirm the blood glucose lowering effect of Tai chi in type-2 DM patients.

However, according to the results of this study, compared with nonexercise, Tai chi had significant decreasing effect on FBG, HbA1c, and 2hPBG among type-2 DM patients, indicating that Tai chi had a positive effect for diabetics. Theoretically, as a regular small- and medium-intensity aerobic exercise, Tai chi can promote the metabolism of cells and tissues, promote blood back-flowing to the heart, improve body's utilization of glucose, increase target cell reactivity, improve body's glucose tolerance, prevent the composition of HbA1c, and accelerate the combination of hemoglobin and oxygen. Therefore, the blood glucose can be further controlled, thereby reducing the levels of FBG, HbA1c, and 2hPBG.

From the results of this study, compared with other aerobic exercise, including running, walking, and dancing, Tai chi did not show a better effect on blood glucose control in type-2 DM patients, which was consistent with the results published by Lee et al. However, in the case of reducing HbA1c, this study showed that Tai chi had some advantages over other aerobic exercise, but the difference was at the margin of statistical significance (*P* = 0.05). Relevant studies, including our previous study, have found that mind-body exercises had the effect of reducing HbA1c in type-2 DM patients. One study showed that walking combined with meditation exercise had a better effect on HbA1c regulation than simple walking [29]; the results of our previous meta-analysis also showed that, compared with other aerobic exercise, Qigong exercise had more advantages in downregulating HbA1c level of type-2 DM patients [30]. Therefore, we considered that Tai chi as an exercise with the combination of movement and meditation could suppress sympathetic activation, thus can improve glycemic control as neutrally mediated vasoconstriction, and in the meanwhile reduce glucose delivery and uptake in skeletal muscle [31].

At present, there were few studies on whether there was dose-response relationship between physical exercise, including Tai chi, with FBG and HbA1c control in type-2 DM patients. However, a recent study showed that there was curvilinear dose-response relationship between HbA1c and total exercise amount, higher exercise amount was associated with lower HbA1c level [32]. According to the results of this study, for both FBG and HbA1c, higher exercise amount subgroup corresponded to lower effect size in a certain range. Therefore, we believed that there was a certain dose-response relationship between Tai chi with FBG and HbA1c control in type-2 DM patients, while the authenticity needs to be verified by more rigorous clinical trials or meta-analysis.

According to meta-analysis and the included studies, total Tai chi exercise amount of 4000–5999 minutes could result in better blood glucose and HbA1c control. For type-2 DM patients, to regulate blood glucose and HbA1c levels, Tai chi exercise of 80–120 minutes per week for more than one year or 160–240 minutes per week for more than a half year was recommended. This recommended Tai chi exercise amount was basically the same as the 150-minute moderate-intensity exercise per week mentioned in most current clinical practice guidelines [33].

There are limitations to this study that must be considered. Firstly, due to limited conditions, only PubMed, The Cochrane Library, EM base, SCI, CNKI, Wan Fang Data, and VIP were searched, and there might be omissions; secondly, the quality of included papers was low, the random distribution method and blind method was not described, some trials failed to indicate whether there was missing subject, only indicating random distribution, and the modified Jadad score of most trials was 2; thirdly, the heterogeneity of included studies was quite large, only random-effect model could be adopted, which had certain impact on the results; fourthly, there was a large possibility of publication bias among included trials.

## 5. Conclusion

Tai chi can effectively affect the management of blood glucose and HbA1c in type-2 DM patients. Long-term adherence to Tai chi has a better role in reducing blood glucose and HbA1c levels in type-2 DM patients.

## Figures and Tables

**Table 1:** Characteristics and quality assessments of the included trials.

First author, year	Patients (total, E/C)	Age (mean (SD), E/C)	Experimental intervention	Control	Duration, sessions with supervision per week, time per session
Yan 2004	18, 10/8	51.8 (7.4)/53.1 (7.8)	Tai chi	N	24 weeks, 7, 40 min
Yu 2004	40, 20/20	50 (58)/49 (5.6)	Tai chi	N	12 weeks, 7, 60 min
Jiang 2007	36, 18/18	57.2 (4.2)/55.0 (6.2)	Tai chi	N	20 weeks, 7, 40 min
Tracey T. 2007	38, 18/20	65 (7.8)	Tai chi	N	16 weeks, 2, 60 min
Paul Lam 2008	53, 28/15	63.2 (8.6)/60.7 (12.2)	Tai chi	N	24 weeks, 2, 60 min
Ying Zhang 2008	20, 10/9	57.4 (6.2)	Tai chi	N	14 weeks, 5, 60 min
Wang 2009	54, 28/26	49.21 (11.12)/48.83 (10.54)	Tai chi	Social dancing exercise	24 weeks, 5, 30 min
Wu 2010	40, 20/20	NR	Tai chi	N	24 weeks, 3, 60 min
Shih-Chuen Chen 2010	104, 56/48	59.1 (6.2)/57.4 (5.8)	Tai chi	Aerobic exercise	12 weeks, 3, 60 min
Li X. 2013	60, 30/30	57.3 (10.3)	Tai chi	N	8 weeks, 7, 45 min
Li Z. 2013	87, 43/44	54.21 (9.47)/52.69 (8.37)	Tai chi	N	12 weeks, 7, 30 min
Sununta 2013	64, 32/32	NR	Tai chi	N	12 weeks, 5, 30 min
Tang 2014	28, 14/14	61.40 (5.08)/61.53 (6.23)	Tai chi	N	24 weeks, 5, 15 min
Tang 2014	29, 15/14	62.73 (7.17)/61.53 (6.23)	Tai chi	N	24 weeks, 5, 30 min
Tang 2014	27, 13/14	59.40 (6.68)/61.53 (6.23)	Tai chi	N	24 weeks, 5, 45 min
Zhang 2015	100, 50/50	55.36 (2.78)/61.53 (6.23)	Tai chi	N	4 weeks, 7, 30 min

E: experimental group; C: control group; N: nonexercise; NR: not reported; min: minute; SD: standard deviation.

**Table 2:** The quality evaluation of the methodology.

Inclusion research	Randomization	Concealment of allocation	Double blinding	Withdrawals and dropouts	Jadad score
Yan 2004	Yes	No	No	No	2
Yu 2004	Yes	No	No	No	2
Jiang 2007	Yes	No	No	No	2
Tracey T. 2007	Yes	No	Double blind	Yes	5
Paul Lam 2008	Yes	Computer generation	Double blind	Yes	6
Ying Zhang 2008	Yes	No	No	Yes	3
Wang 2009	Yes	No	No	Yes	3
Wu 2010	Yes	No	No	No	2
Shih-Chuen Chen 2010	Yes	No	No	Yes	3
Li X. 2013	Yes	No	No	No	2
Li Z. 2013	Yes	No	No	Yes	3
Sununta 2013	Yes	No	Double blind	Yes	5
Tang 2014	Yes	No	No	Yes	3
Zhang 2015	Yes	No	No	No	2
